# Benznidazole-Associated Rash in the Setting of Incorrectly Diagnosed Chronic Chagas Disease

**DOI:** 10.4269/ajtmh.24-0790

**Published:** 2025-02-25

**Authors:** Norman L. Beatty, Priya P. Patel, Rodrigo F. Alcala

**Affiliations:** ^1^ Division of Infectious Diseases and Global Medicine, Department of Medicine, University of Florida College of Medicine, Gainesville, Florida;; ^2^ Emerging Pathogens Institute, University of Florida, Gainesville, Florida;; ^3^ University of Florida College of Medicine, Gainesville, Florida

**Figure 1.**
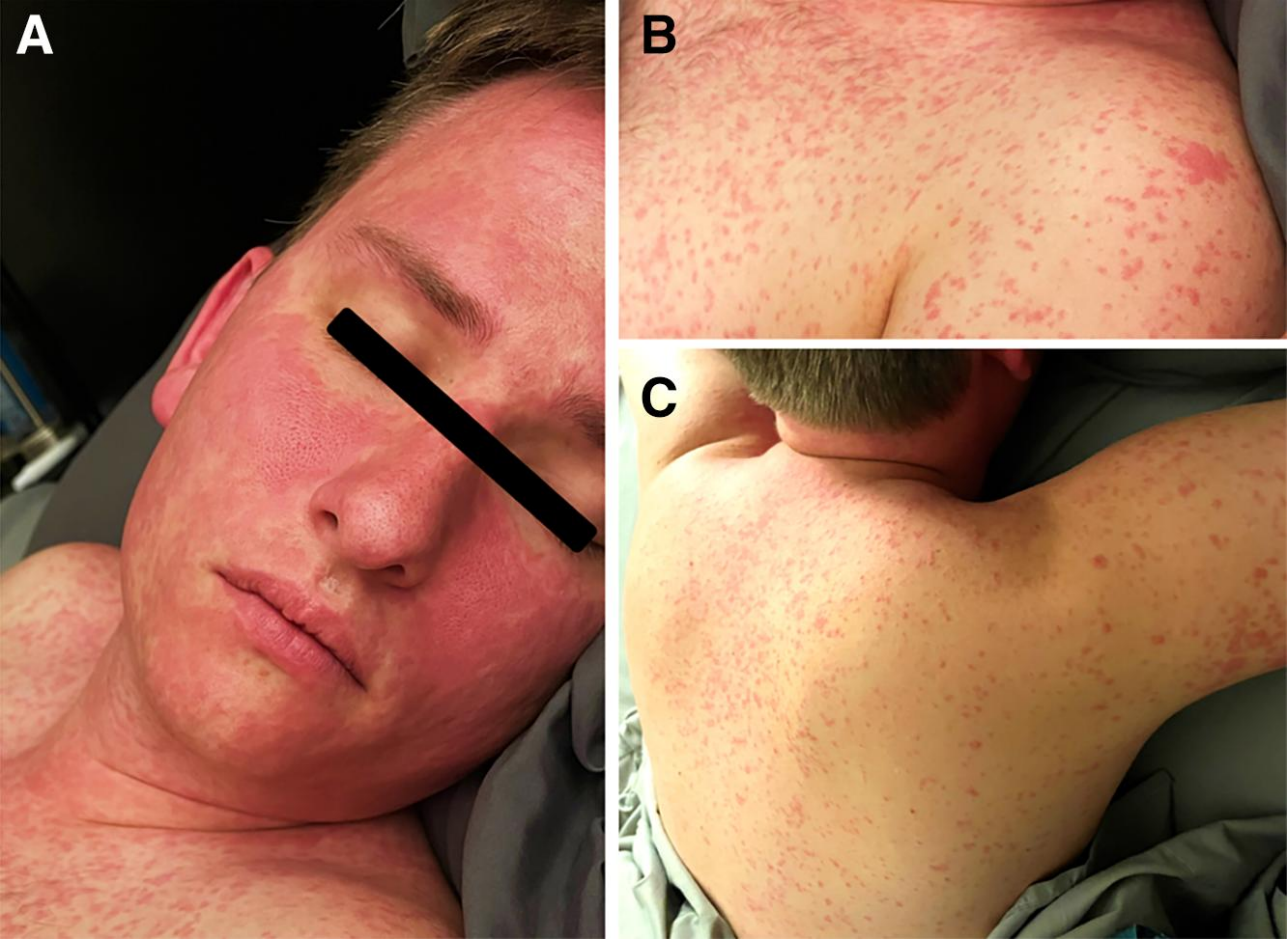
Blanching erythematous maculopapular rash throughout the body due to a cutaneous adverse drug reaction causing maculopapular exanthema from benznidazole use. (**A**) Head and neck with coalescing raised rash largely sparing the orbits, with innumerable raised papules on the body, as seen on the (**B**) chest and (**C**) back.

A 25-year-old man with no past medical history presented to the travel medicine and tropical diseases clinic at the University of Florida Health as an urgent outpatient consult with concerns about chronic Chagas disease (CD). He had recently donated blood and received a letter in the mail indicating that he had screened positive for CD. The patient visited his primary care provider, who started him on oral benznidazole 500 mg, divided into two daily doses, after reading an online recommendation to administer a dose of 5 mg/kg/day. No additional CD testing was conducted. Within 7 days, he developed generalized malaise accompanied by a diffuse erythematous maculopapular urticarial rash throughout his body (Figure 1). A detailed history taken in our clinic to assess epidemiological risk for CD revealed that the patient and his mother were born in the state of Utah. He had never received a blood transfusion or transplanted organ. The patient lived in the southern third of Chile from 2015 to 2017 while on a service mission trip, where CD is nonendemic.^1^ He currently lives in a suburban neighborhood in north Florida. He has no known exposure to the triatomine (kissing bug) in either Chile or Florida. A review of systems was negative aside from the diffuse, itchy, blanching, maculopapular rash. His lips and tongue did not swell, and he did not experience wheezing or breathing problems. His vitals and physical examination were otherwise normal. An electrocardiogram and chest X-ray were negative for abnormalities. Rapid lateral flow CD testing (Chagas Detect™ Plus, InBios, Seattle, WA) results were negative. Benznidazole was immediately discontinued, and oral prednisone 60 mg daily was initiated. Additional serum samples were sent to two reference laboratories to investigate anti-*Trypanosoma cruzi* (*T. cruzi*) IgG antibodies (Weiner Chagatest Recombinante v.3.0, Hemagen Chagas’ Test, Columbia, MD). Both assays returned negative results within 5 days. The complete metabolic panel and blood counts were stable.

The patient was advised that he does not have evidence of chronic CD, given the negative results of three FDA-cleared serological assays and the absence of epidemiological concerns for *T. cruzi* transmission.^2^ Our patient was diagnosed with maculopapular exanthema (MPE) due to an adverse reaction to benznidazole. A 3-week oral prednisone taper was completed, resulting in the resolution of symptoms. Dermatological reactions are commonly reported among those taking benznidazole, ranging from MPE to severe cutaneous adverse reactions, including Stevens–Johnson syndrome and toxic epidermal necrolysis.^3^^–^^5^ Other common side effects in those taking benznidazole include headache, extremity neuropathy, nausea, poor appetite, and transaminitis.^4^^,^^5^ Diagnostic guidelines are available to help healthcare providers make a serological diagnosis of chronic CD.^2^^,^^3^ A serological diagnosis of chronic CD involves the detection of anti-*T. cruzi* antibodies using two separate assays that employ different *T. cruzi* antigenic profiles.^2^^,^^3^ Blood donor CD screening in the U.S. is highly sensitive, but false positive results can occur. Anyone who screens positive after blood donation should consult a healthcare provider with experience in the diagnosis and management of chronic CD to avoid misdiagnosis and inadvertent adverse reactions, as described in our patient.^1^^,^^2^
